# An algorithm to discover gene signatures with predictive potential

**DOI:** 10.1186/1756-9966-29-120

**Published:** 2010-09-02

**Authors:** Robin M Hallett, Anna Dvorkin, Christine M Gabardo, John A Hassell

**Affiliations:** 1 Department of Biochemistry and Biomedical Sciences, Centre for Functional Genomics, McMaster University, 1200 Main Street West, Hamilton, Ontario, L8N 3Z5 Canada

## Abstract

**Background:**

The advent of global gene expression profiling has generated unprecedented insight into our molecular understanding of cancer, including breast cancer. For example, human breast cancer patients display significant diversity in terms of their survival, recurrence, metastasis as well as response to treatment. These patient outcomes can be predicted by the transcriptional programs of their individual breast tumors. Predictive gene signatures allow us to correctly classify human breast tumors into various risk groups as well as to more accurately target therapy to ensure more durable cancer treatment.

**Results:**

Here we present a novel algorithm to generate gene signatures with predictive potential. The method first classifies the expression intensity for each gene as determined by global gene expression profiling as low, average or high. The matrix containing the classified data for each gene is then used to score the expression of each gene based its individual ability to predict the patient characteristic of interest. Finally, all examined genes are ranked based on their predictive ability and the most highly ranked genes are included in the master gene signature, which is then ready for use as a predictor. This method was used to accurately predict the survival outcomes in a cohort of human breast cancer patients.

**Conclusions:**

We confirmed the capacity of our algorithm to generate gene signatures with *bona fide *predictive ability. The simplicity of our algorithm will enable biological researchers to quickly generate valuable gene signatures without specialized software or extensive bioinformatics training.

## Introduction

Clinicians are commonly faced with two important decisions when treating cancer patients: whether or not adjuvant chemotherapy is required, and selecting the most appropriate treatment. Traditionally, several histopathological characteristics of the tumor are taken into consideration when deciding on the best treatment[1]. However, it has been reported that 70-80% of breast cancer patients do not benefit from the use of chemotherapy, but are still exposed to the deleterious side effects of these drugs[2]. Therefore additional prediction methods are needed to improve the quality of life for breast cancer patients. One of these methods relies on gene expression profiling based predictors, which can be used to further inform the decision making process and increase a clinician's ability to successfully treat cancer patients [3]. Recently, researchers developed a 70-gene signature that can correctly separate patients into good- and poor-prognosis groups, and identified patients who can be spared unnecessary chemotherapy [2,4]. However, constructing such a signature requires the use of various clustering and classification algorithms, which in turn require specialized software and bioinformatics training. Consequently, the need arises for strategies that can be used to generate predictive gene signatures, which are amenable to the software and skill sets available to the cancer biologist.

Typically gene expression based predictors are "trained" on a cohort of samples whose gene expression profiles are known, and for which at least one biological characteristic has been measured[5]. After the "training" of a predictor it must be validated on a set of samples, which were not used to initially "train" the algorithm. Predictors should in turn be able to accurately forecast the biological characteristic of samples of interest.

For our purposes we used a data set consisting of whole tumor gene expression profiles derived from 295 primary human breast tumors, as well as clinical data relating to the patients survival and occurrence of metastasis [2]. We then coarsely grained the expression data into high, average and low expression levels, and ranked genes based on the extent of their expression in patients who either survived or succumbed to breast cancer. In this fashion we were able to find genes whose transcripts generally had high and low expression in patients who succumbed and survived, respectively, and vice versa. By combining the top ranked candidates from a 144 patient training dataset we were able to create a 20 gene signature which performed well on a 151 patient validation dataset.

Our analyses establish an effective method to obtain gene expression based predictors that clearly separate human breast cancer patients into distinguishable prognosis groups with statistically significant differences in survival.

## Methods

### Microarray and clinical data

The microarray data used for our analyses was obtained from the Stanford microarray repository (downloaded from http://microarray-pubs.stanford.edu/wound_NKI/explore.html, henceforth called NKI dataset). A matrix containing clinical data for the patients that provided samples for the microarray profiles used in the present study was downloaded from the same location. This data consists of the gene expression profiles of primary breast tumors biopsied from 295 human breast cancer patients. All patients had either stage I or stage II breast cancer, and were younger than 53 years old. The prevalence of lymph-node positive and lymph-node negative disease was 49% and 51%, respectively. We combined these data into one matrix containing indices for survival, metastasis, and the gene expression profiles for each patient. We used 12 year overall survival as the clinical endpoint for this study.

### Organization of data

We blindly divided the patients into two groups consisting of similar numbers of patients, one for algorithm training (144 patients) and the other for algorithm validation (151 patients).

### Defining levels of gene expression

In order to rank the predictive ability of a gene, we first needed to assess its expression in each given patient tumor relative to its expression in the tumors of all patients. To this end we first calculated the 95% confidence interval for expression of each gene. The level of expression for each gene was then defined as the following:

i) If the expression of a gene in a given patient's tumor was greater than the upper limit of the 95% confidence interval for the expression of the same gene across all patient tumors, then the gene's expression was scored high for that patient's tumor.

ii) If the expression of a gene in a given patient's tumor was less than the lower limit of the 95% confidence interval for the expression of the same gene across all patient tumors, then the gene's expression was scored low for that patient's tumor.

iii) If the expression of a gene in a given patient's tumor was within the 95% confidence interval for the expression of the gene across all patient tumors, then the gene's expression was scored average for that patient's tumor. These steps were completed for every gene across every patient tumor.

This new matrix consisting of clinical patient data, as well as the gene expression score for each gene, represented by either high, average or low, was then used to rank the genes based on their predictive capacity.

### Ranking the predictive capacity of each gene

We ranked each gene in the training set according to its expression in the tumor of patients who either survived or died from breast cancer. We expected genes whose expression was associated with poor prognosis to be generally highly expressed in patients who died and to be expressed at low levels in patients who survived. Conversely, we expected genes whose expression was associated with good prognosis to generally be highly expressed in patients who survived and to be expressed at low levels in those patients who succumbed. Therefore, the ranking of the genes was performed as follows for genes predictive of poor or good prognosis.

### Genes predictive of poor prognosis

i) A predictive score for each gene was computed for each gene across all patients, and was initially set at 0.

ii) 1. The score for each gene was increased by 1 when the patient had both high gene expression and died, or had both low gene expression and survived.

2. The score was decreased by 1 when the patient had both low gene expression and died, or had both high gene expression and survived.

3. Average gene expression levels did not lead to any changes in the predictive score.

### Genes predictive of good prognosis

i) A predictive score for each gene is computed for each gene across all patients, and was initially set at 0.

ii) 1. The score was increased by 1 when the patient had both high gene expression and survived, or had both low gene expression and died.

2. The score is decreased by 1 when the patient had both low gene expression and survived, or had both high gene expression and died.

3. Average gene expression levels did not lead to any changes in the predictive score.

We then combined the top ranked genes from both the poor-prognosis and good-prognosis gene lists to generate a predictor gene signature. We completed all of the steps described above using Microsoft Excel™ 2007. Template file available upon request.

### Measuring the predictive ability of the gene signature

In order to separate the training data set into good prognosis and poor prognosis groups we summed the expression of both poor-prognosis genes (poor-prognosis gene score) and good-prognosis genes (good-prognosis gene score) for all the patients in our training set. To give each patient a single overall-prognosis score we subtracted the good-prognosis gene score from the poor-prognosis gene score, and ranked the patients according to this new total. This led patients with the highest and lowest expression of poor-prognosis and good-prognosis genes, respectively, to receive the highest scores, and patients with the lowest and highest expression of poor-prognosis and good-prognosis genes, respectively, to receive the lowest scores. In this fashion, high scores were indicative of poor prognosis and low scores were indicative of good prognosis. In order to determine a optimal cut-off score which would yield prognosis predictions with the highest possible specificity and sensitivity, we used receiver-operator characteristic curves (ROC) [6]. This generated a list of possible cut-off scores, as well as each score's associated specificity and sensitivity. We next summed the specificity and sensitivity for each cut-off score and used the cut-off which yielded the highest total. For the random control sample, we generated a 20-gene signature where the signature was populated with randomly selected genes selected by a random number generator http://www.random.org.

### Analysis of survival differences between good-prognosis and poor-prognosis groups

Unless otherwise indicated, GraphPad Prism 5™ software was used to complete survival analysis, linear regression, and comparison of survival means, as well as all associated statistical tests, and ROC analysis, to measure the predictive ability of the prognosis gene signature in both the training and validation data sets. Additional details available as supplementary methods.

### Comparison of models

We calculated the predictive accuracy (Cases correctly predicted Vs All cases), specificity (Cases of correctly predicted good overall survival Vs Cases of actual good overall survival), and positive predictive value (PPV) (Cases correctly predicted of poor survival Vs All cases predicted poor survival) for our 20-gene signature, the Aurora kinase A, and 70-gene signature models. Patients were divided into good and poor survival groups based on Aurora kinase A expression, where the average expression of Aurora kinase A for all patients was used as the cut-off separating the two groups. The 70-gene signature classification for the patients was included in the original clinical data file.

### Gene ontology

Gene names were uploaded to the gene ontology website http://www.geneontology.org, and the biological processes associated with the human form of the gene were recorded.

## Results

### Generation and validation of a gene signature that predicts human breast cancer patient survival

To establish a gene signature that could accurately predict the survival outcome of human breast cancer patients we used a 295 patient database containing both clinical data relating to patient survival and occurrence of metastases, as well as the patient's individual tumor gene expression profiles. We divided this database into training and validation groups, containing 144 and 151 patients, respectively. We then identified genes whose expression levels correlated with patient survival as described in Methods. The 10 most highly ranked genes predictive of poor-prognosis and those 10 genes most highly predictive of good-prognosis established a 20-gene expression based predictor (Table 1).

**Table 1:** Genes comprising the 20-gene signature

				95% CI interval	
**Gene ID#**	**Systemic_name**	**Gene name/symbol**	**Average**	**Upper**	**Lower**

10855	D43950	KIAA0098	-0.004	0.027	-0.035

19769	U96131	TRIP13	-0.039	-0.001	-0.077

14841	NM_014865	KIAA0159	-0.007	0.029	-0.044

15318	Contig55725_RC		-0.219	-0.150	-0.289

12548	AF047002	ALY	-0.040	-0.008	-0.072

3342	NM_004111	FEN1	-0.028	0.003	-0.058

3493	NM_004153	ORC1L	0.037	0.057	0.017

8204	NM_004631	LRP8	0.038	0.067	0.009

3838	NM_002794	PSMB2	-0.024	0.004	-0.051

3938	Contig55771_RC		-0.047	-0.005	-0.088

6615	NM_004496	HNF3A	-0.216	-0.120	-0.312

8786	NM_006113	VAV3	-0.170	-0.107	-0.232

18817	AL161983		-0.015	0.007	-0.037

17540	NM_016613	LOC51313	-0.002	0.022	-0.026

1723	AL133074		-0.078	-0.033	-0.123

23117	Contig14284_RC		-0.324	-0.209	-0.440

57	Contig56678_RC		-0.205	-0.135	-0.274

18904	NM_000125	ESR1	-0.312	-0.215	-0.409

6709	Contig57480_RC	LOC51028	-0.021	0.009	-0.051

6105	NM_005113	GOLGA5	-0.046	-0.024	-0.067

**Figure 1:**
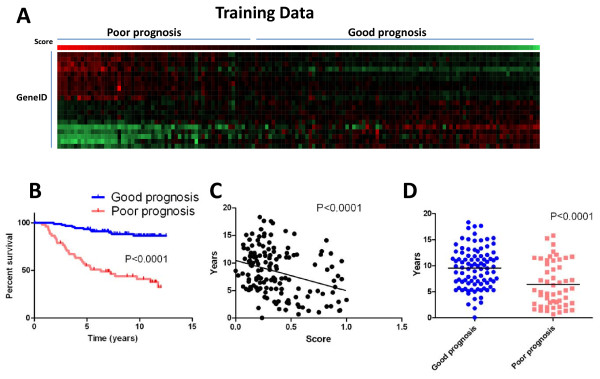
**Our 20-gene signature separates the training data set into poor-prognosis and good-prognosis groups (A, red = high expression, green = low expression) with differences in survival (B), a negative correlation between prognosis score and survival time (C) and differences in mean survival time (D)**.

To learn whether this gene signature could accurately predict survival of the patients from which it was created, we used our 20 gene signature to rank all 144 patients within the training set and divided them into a poor-prognosis group and good-prognosis group (Fig. 1A). We also compared the overall survival between the two groups (Fig. 1B, log-rank test[7], p < 0.0001), fitted linear regression to examine the correlation between time-to-death or censure and prognosis score (Fig. 1C, F-test, significant negative correlation, p < 0.0001), and mean survival time (or time to censure) between the two groups (Fig. 1D, Mann-Whitney test, p < 0.0001). In total, our results demonstrated the capacity of our gene signature to properly segregate human breast cancer patients into good- and poor-prognosis groups.

**Figure 2:**
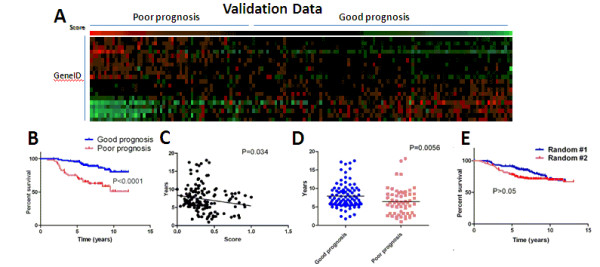
**Our 20-gene signature separates the validation data set into poor-prognosis and good-prognosis groups (A, red = high, green = low) with differences in survival (B), negative correlation between prognosis score and survival time (C), and differences mean survival time (D)**. E) A randomly generated 20-gene signature does not correlate prognosis score to patient survival.

To validate our signature in patients whose data had not been used to generate the signature, we divided the 151 patient validation group into poor-prognosis and good-prognosis groups (Fig. 2A). Again, our signature correctly separated patients based on survival (Fig. 2B, log-rank test p < 0.0001), correlated prognosis score with survival time (Fig. 2C, F-test, significant negative correlation, p = 0.034), and predicted mean survival time (Fig. 2D, Mann-Whitney test, p = 0.0056). To rule out the possibility that our signature's significance was a result of chance, we randomly generated a different 20-gene signature. As expected the random 20-gene signature did not separate patients into groups with differences in survival (Fig. 2E).

### Analysis of the 20-gene signature

To ensure that our algorithm produced predictors with comparable predictive power to other forms of feature selection we compared the 20-gene signature to a previously published Aurora kinase A expression model, as well as the FDA approved 70-gene signature (MammaPrint™) [2,8]. The 70-gene MammaPrint signature was originally tested on the NKI dataset, the same dataset we used for the development of our 20-gene signature. We also included the Aurora kinase A expression model, as this model was shown to predict breast cancer patient outcome with similar accuracies to many other feature selection techniques [8]. Our 20-gene signature had a slightly higher predictive accuracy (0.67 Vs 0.64 Vs 0.61, 20-gene signature, Aurora kinase A, 70-gene signature models, respectively), and roughly comparable specificity and positive predictive value to the Aurora kinase A expression and 70-gene signature models (Table 2). Importantly, these comparisons indicate that our algorithm produces classifiers of at least similar predictive power than those produced by other feature selection techniques.

**Table 2:** Predictive ability of the Aurora kinase A, 20-gene signature, and 70-gene signature.

Test	Aurora kinase A (NKI dataset)	20-gene (151 validation set)	70-gene (NKI dataset)
Predictive accuracy	0.64	0.67	0.61

Specificity	0.6	0.66	0.66

Positive predictive value	0.4	0.38	0.4

Since gene signatures are readily measurable cell characteristics which serve to indicate biological processes, we mapped the gene ontology of each gene-member of our 20-gene signature to learn whether our signature was linked to a particular biological process (Table 3). We found that genes linked to poor-prognosis were generally involved in processes such as mitosis, transcription, as well as DNA replication and DNA repair, whereas genes linked to good-prognosis were generally involved in processes such as cell differentiation and induction of apoptosis. These observations are consistent with the histological observations that patients with highly proliferative and poorly differentiated tumors generally have poorer survival outcomes than those with well differentiated and non-proliferative tumors.

**Table 3:** Gene ontology of the 20-gene signature

ID#	Systemic_name	Symbol	Biological Process
10855	D43950	KIAA0098	Protein folding/Response to virus

19769	U96131	TRIP13	Double stranded break DNA repair/Meosis I/Spermatogonial Development/Oocyte maturation/Pachytene (cell cycle)/Meotic recombination/Transcription from RNA Pol II promoter

14841	NM_014865	KIAA0159	N/A

15318	Contig55725_RC		N/A

12548	AF047002	ALY	Interspecies interaction between organisms/Intronless viral mRNA export from nucleus/mRNA export from nucleus/mRNA processing/Transport

3342	NM_004111	FEN1	DNA repair/DNA replication/Double stranded break DNA repair/UV protection/Phosphoinositide mediated signaling

3493	NM_004153	ORC1L	DNA replication/DNA dependent DNA replication

8204	NM_004631	LRP8	Cytokine mediated signaling pathway/Endocytosis/Hippocampus development/Layer formation in the cerebral cortex/Lipid metabolic process/Positive regulation of kinase activity/Proteolysis/Signal transduction

3838	NM_002794	PSMB2	Anaphase-promoting complex-dependent proteosomal ubiquitin dependent protein catabolic process/Interspecies interaction between organisms/Negative regulation of ubiquitin ligase activity involved in mitotic cell entry/Positive regulation of ubiquitin ligase activity involved in mitotic cell entry/Proteolysis involved in cellular protein catabolic process

3938	Contig55771_RC		N/A

6615	NM_004496	HNF3A	Branching morphogenesis of a tube/Chromatin remodelling/Epithelial cell differentiation/Prostate gland development/Glucose homeostasis/Hormone metabolic process/Lung development/Multicellular organismal development/Negative regulation of survival gene product/Negative regulation of transcription fro RNA pol II promoter/Neuron fate specification

8786	NM_006113	VAV3	Angiogenesis/Apoptosis/Cell Migration/Induction of apoptosis by extracellular signals/Integrin mediated signaling pathway/Lamellipodium assembly/Positive regulation of cell adhesion/Positive regulation of PI3 kinase activity/Regulation of GTPase activity/Regulation of Rho protein signal transduction/Small GTPase mediated signal transduction/Vesicle fusion

18817	AL161983		Regulation of translation/Translation initiation

17540	NM_016613	LOC51313	N/A

1723	AL133074		Apoptosis/Cell cycle arrest/Induction of apoptosis/Response to stress

23117	Contig14284_RC		N/A

57	Contig56678_RC		N/A

18904	NM_000125	ESR1	Androgen metabolic process/Antral ovarian follicle growth/Epithelial cell development/Epithelial cell proliferation involved in mammary gland duct elongation/Estrogen receptor signaling pathway/Male gonad development/Mammary gland alveolus development/Mammary gland branching involved in pregnancy/Neuroprotection/Osteoblast development

6709	Contig57480_RC		N/A

6105	NM_005113	GOLGA5	Golgi organisation/Golgi vesicle transport/Protein amino acid phosphorylation/Retrograde transport, vesicle recycling within golgi

## Discussion

We sought to generate an algorithm with the following properties: (i) simple implementation with straight forward methodology, and (ii) high predictive accuracy. The reasons for this were to facilitate non-bioinformatic expert biologist development of valuable and biologically useful gene expression based prediction models. Importantly, we completed all steps of our algorithm using Microsoft Excel™ 2007, and will share the template files used for these analyses with interested researchers. This software is widely (if not universally) accessible to and used by the biological research community, suggesting that implementation of this technique will not be hampered by lack of software or training. As mentioned previously, most other feature selection techniques require the use of sophisticated clustering and classification algorithms, whose use requires specialized software and software based training.

To confirm that our algorithm produced a prediction model with comparable predictive power to other techniques in feature selection we compared its predictive power with that of an Aurora kinase A expression model as well as the 70-gene signature MammaPrint™ model. The Aurora kinase A model was previously shown to have comparable predictive accuracy to several feature selection techniques at predicting breast cancer patient survival, and can be used to make comparisons between feature selection techniques [8]. Additionally, the 70-gene signature has previously been tested on the NKI dataset, which allowed us to make model comparisons on the same patients. The 70-gene signature is also used clinically and thus represents a "gold standard" against which to compare predictive accuracy of gene signatures which predict breast cancer patient outcome [9]. We observed that our model had a slightly higher overall predictive accuracy than either the Aurora kinase A expression model or the 70-gene signature, and all three models had comparable specificities and positive predictive values (Table 2). Importantly, these observations demonstrate that our algorithm produces prediction models with comparable accuracy to other feature selection techniques while having generally better accessibility and useability for biological research scientists. To this end, we've begun using our algorithm to generate gene expression based prediction models of breast cancer cell sensitivity to commonly used anti-cancer therapies.

## Conclusion

Here, we present an algorithm to generate gene signatures with predictive potential. It is noteworthy that our algorithm was developed using Microsoft Excel™ and tested using GraphPad Prism5™, commonly available software that should significantly increase its use. Importantly, the signature developed using our method had comparable predictive accuracy to either the Aurora kinase A expression or 70-gene MammaPrint™ models [2,8]. Our methods represent a novel and broadly applicable technique to generate predictive gene signatures that we anticipate will prove useful to the molecular biological research community.

## Conflict of interests

The authors declare that they have no competing interests.

## Authors' contributions

RMH, conception of project; RMH, AD, CMG, performed research; RMH, AD, CMG, JAH, interpretation of data and writing of manuscript.All authors have read and approved the final manuscript.

## Appendix 1

### Supplementary methods

#### Survival analysis

Survival analysis was completed using Graphpad Prism 5™ software's "survival" option. Time to endpoint or time to study censorship was included as the independent variable (x-axis column) and death or survival (denoted 1 = death, 0 = survival) was included on the y-axis column. Independent y-axis columns were used for each group (good or poor prognosis). Statistical analyses (Log-rank test) was accessed and completed using the Graphpad analyze tab.

#### Linear regression

Linear regression was completed using Graphpad Prism 5™ software's "XY" option. The survival score was plotted as the independent variable (x-axis column), whereas survival time or time to death was plotted in the y-axis column. Statistical analyses to confirm correlation was completed using the Graphpad analyze tool.

#### Survival time mean

Survival time mean comparison was completed using Graphpad Prism 5™ software's "column" option. The survival or time to death times for both the good and poor prognosis groups were plotted in independent columns. A t-test was used to compare the means between the groups, and was completed using the Graphpad analyze tool.

#### ROC analysis

The ROC analysis to determine optimal cut-off score was complete using Graphpad Prism 5™ software's "column" option. The survival scores for the good and poor outcome groups were plotted in independent columns. The ROC analysis tool (accessed through the Graphpad analyze tool) was used determined the sensitivity and specificity of each possible cut-off score. The cut-off score yielding the highest sum of specificity and sensitivity was then used to divide the patients into good and poor outcome groups.
